# Genetic Interaction between *Tmprss2-ERG* Gene Fusion and *Nkx3*.*1*-Loss Does Not Enhance Prostate Tumorigenesis in Mouse Models

**DOI:** 10.1371/journal.pone.0120628

**Published:** 2015-03-17

**Authors:** Douglas E. Linn, Roderick T. Bronson, Zhe Li

**Affiliations:** 1 Division of Genetics, Brigham and Women’s Hospital, Boston, Massachusetts 02115, United States of America; 2 Department of Medicine, Harvard Medical School, Boston, Massachusetts 02115, United States of America; 3 Rodent Histopathology, Harvard Medical School, Boston, Massachusetts 02115, United States of America; Innsbruck Medical University, AUSTRIA

## Abstract

Gene fusions involving ETS family transcription factors (mainly *TMPRSS2-ERG* and *TMPRSS2-ETV1* fusions) have been found in ~50% of human prostate cancer cases. Although expression of *TMPRSS2-ERG* or *TMPRSS2-ETV1* fusion alone is insufficient to initiate prostate tumorigenesis, they appear to sensitize prostate epithelial cells for cooperation with additional oncogenic mutations to drive frank prostate adenocarcinoma. To search for such ETS-cooperating oncogenic events, we focused on a well-studied prostate tumor suppressor *NKX3*.*1*, as loss of *NKX3*.*1* is another common genetic alteration in human prostate cancer. Previous studies have shown that deletions at 8p21 (harboring *NKX3*.*1*) and 21q22 (resulting in *TMPRSS2-ERG* fusion) were both present in a subtype of prostate cancer cases, and that ERG can lead to epigenetic silencing of *NKX3*.*1* in prostate cancer cells, whereas NKX3.1 can in turn negatively regulate *TMPRSS2-ERG* fusion expression via suppression of the *TMPRSS2* promoter activity. We recently generated knockin mouse models for *TMPRSS2-ERG* and *TMPRSS2-ETV1* fusions, utilizing the endogenous *Tmprss2* promoter. We crossed these knockin models to an Nkx3.1 knockout mouse model. In *Tmprss2-ERG;Nkx3.1^+/-^* (or *^-/-^*) male mice, although we observed a slight but significant upregulation of *Tmprss2-ERG* fusion expression upon *Nkx3*.*1* loss, we did not detect any significant cooperation between these two genetic events to enhance prostate tumorigenesis *in vivo*. Furthermore, retrospective analysis of a previously published human prostate cancer dataset revealed that within *ERG*-overexpressing prostate cancer cases, *NKX3*.*1* loss or deletion did not predict biochemical relapse after radical prostatectomy. Collectively, these data suggest that although *TMPRSS2-ERG* fusion and loss of *NKX3*.*1* are among the most common mutational events found in prostate cancer, and although each of them can sensitize prostate epithelial cells for cooperating with other oncogenic events, these two events themselves do not appear to cooperate at a significant level *in vivo* to enhance prostate tumorigenesis.

## Introduction

*ETS* gene fusions are prevalent in about half of human prostate cancer cases, one of the most common malignancies among Western males [1,2]. Coding regions of several ETS family transcription factors (e.g., ERG, ETV1) are often rearranged to control regions of androgen-responsive genes, particularly the *TMPRSS2* gene, leading to aberrant expression of *ETS* genes. To address the role of *ETS* fusions in prostate cancer, several transgenic mice have been generated that ectopically express ERG or ETV1 from the *Probasin* (*PB*) promoter (*PB-ERG* or *PB-ETV1*) [3–6]. Depending on the strain background and splicing variants of *ETS* genes, some of these studies suggested that there are Prostate Intraepithelial Neoplasia (PIN)-like lesions in *PB-ERG* and *PB-ETV1* transgenic males [4–7], whereas others indicated that *PB-ERG* transgenic males are normal in their prostates [3,8]. We recently reported mouse models of prostate cancer that recapitulate the most frequent *ETS* gene fusions, *TMPRSS2-ERG* and *TMPRSS2-ETV1*, with ectopic ERG or ETV1 expression from the endogenous *Tmprss2* promoter [9]. We found that prostates from either *Tmprss2-ERG* (*T-ERG*) or *Tmprss2-ETV1* (*T-ETV1*) knockin male mice are largely normal. Although both the *ETS* transgenic overexpression models and our *Tmprss2-ETS* knockin models suggest that ectopic expression of ERG or ETV1 alone in murine prostates is not sufficient to initiate prostate tumorigenesis, mouse modeling studies further demonstrated that ectopic ERG or ETV1 expression can cooperate with *Pten*-loss (thus leading to activation of the PI3K pathway) to drive prostate cancer development [8–10]. Consistent with these, in a tissue reconstitution model, lentiviral overexpression of ERG (or ETV1) in prostate cells collaborates with activation of the PI3K pathway or the androgen receptor (AR) pathway to induce distinct prostate carcinomas [11]. These observations suggest that although aberrant expression of ETS factors alone in prostates is insufficient for prostate cancer, it sensitizes prostate epithelial cells for cooperation with additional oncogenic mutations to drive frank prostate adenocarcinoma.

In addition to *ETS* gene fusions and aberrant genetic alterations that activate the PI3K pathway (e.g., *PTEN*-loss), another frequent mutational event in prostate cancer is loss of regions within chromosome 8p21, to which the homeobox gene *NKX3*.*1* maps [12,13]. Strong evidence supports the notion that loss of *NKX3*.*1* is an early event in prostate carcinogenesis, as it occurs in up to 85% of PIN lesions and early invasive cancers [14]. *Nkx3*.*1* is one of the earliest known genes expressed in the developing prostate and subsequent studies have validated its importance in prostate epithelial cell differentiation [14]. Previously expression profiling has defined three subtypes of prostate cancer and among these, the subtype-2 prostate cancer cases, which often exhibit a more aggressive phenotype, have been found to harbor deletions at 8p21 (*NKX3*.*1*) and 21q22 (resulting in *TMPRSS2-ERG* fusion) [13]; thus, loss of *NKX3*.*1* has been predicted to synergize with *TMPRSS2-ERG* fusion to promote prostate tumorigenesis, but this has not been validated experimentally. Furthermore, it has also been reported that ERG could lead to epigenetic silencing of *NKX3*.*1* in prostate cancer cells through induction of the histone methyltransferase EZH2 [15].

While mouse models of *Nkx3*.*1*-loss do not exhibit signs of prostate cancer [16,17], they are hyperplastic in their prostates and display cooperativity with *Pten*-loss for prostate cancer development [18], thus offering a sensitized background to test whether *Tmprss2-ETS* fusions exhibit a similar synergy. To that end, we crossed our *T-ERG* knockin mouse line [9] with a previously characterized *Nkx3*.*1*-null line [16] and analyzed prostate histopathology in aged cohorts. We observed a slight increase in *T-ERG* expression after *Nkx3*.*1*-loss, consistent with a recent report detailing negative regulation of the *TMPRSS2* locus by NKX3.1 [19]. However, this subtle increase in *T-ERG* fusion expression coupled with *Nkx3*.*1*-loss did not promote prostate tumorigenesis. A similar phenotype was observed for our *T-ETV1* model [9] under the complete *Nkx3*.*1*-loss background. Collectively these results suggest that although there is a genetic interaction between *Nkx3*.*1*-loss and *Tmprss2-ERG* gene fusion (to increase the *Tmprss2* promoter activity), this interaction does not enhance prostate cancer development. Our study further highlights the selectivity *TMPRSS2-ETS* fusions have with cooperating mutations.

## Materials and Methods

### Mouse strains, procedures, and tissue preparation

*Tmprss2-ERG* (*T-ERG*) knockin mice and *Pten* knockout (*Pten* ^*+/-*^) mice were generated previously [9]. *Nkx3*.*1* knockout (*Nkx3*.*1* ^*-/-*^) mice were obtained from the Mouse Models of Human Cancers Consortium (MMHCC) repository. All mice were maintained on a mixed genetic background and housed in pathogen-free barrier environment. Mice were sacrificed by carbon dioxide asphyxiation. Prostate tissues used for immunohistochemistry (IHC) were fixed for 16 hours in 10% formalin (Fisher), dehydrated, and embedded in paraffin. Tissues used for immunofluorescent (IF) staining were fixed in 10% formalin (Fisher) for 1 hour, washed in PBS, then saturated in 30% sucrose overnight at 4°C. Tissues were then embedded in OCT compound (Sakura) and stored at-80°C prior to cryosectioning. All mouse experiments and procedures were approved by the Institutional Animal Care and Use Committee (IACUC) of Boston Children’s Hospital where the mice were housed, under the Protocol Number 11–10–2034 (renewed as 14–09–2764R).

### Histology, Immunohistochemistry and Immunofluorescent staining

Paraffin-embedded tissue sections were stained with Hematoxylin and Eosin (H&E) and reviewed by a trained rodent histopathologist. Pathology was defined as previously described [20–22]. Histology summaries are presented as frequency of HG-PIN lesions detected in any prostate lobes (unless otherwise indicated). IHC was carried out by rehydrating sections, followed by performing antigen unmasking with Tris-EDTA buffer. Sections were blocked with 2.5% goat serum for 1 hour at room temperature and incubated with primary antibodies overnight at 4°C. Antibodies for ERG (Epitomics 2805) and Nkx3.1 (Dr. Charles Bieberich, UMBC) were used for IHC. IHC staining was visualized using DAB substrate (Vector Labs) and was counter-stained with hematoxylin. Slides were dehydrated and sealed using Permount mounting media (Fisher). For IF staining, cryosections of prostate tissues were cut at 8μm, blocked in 2.5% goat serum, and incubated with primary antibodies overnight at 4°C. Antibodies for IF were used to detect K5 (Covance PRB-160P) or K8 (Covance MMS-162P). Alexa Fluor-conjugated secondary antibodies (Life Technologies) were incubated for 1 hour at room temperature. Nuclei were counterstained with DAPI and slides were sealed with Vectashield mounting media (Vector Labs). IHC scoring was performed by calculating H-score based on percentage of stained cells and staining intensity [23]. Specifically, 4 fields were chosen at random from each slide at x 400 magnification and the staining intensity in the malignant cell nuclei was scored as 0, 1, 2, or 3 corresponding to the presence of negative, weak, intermediate, and strong brown staining, respectively. The total number of cells in each field and the number of cells stained at each intensity were counted. The average percentage positive was calculated and the following formula was applied: H-score = (% of cells stained at intensity category 1 x 1) + (% of cells stained at intensity category 2 x 2) + (% of cells stained at intensity category 3 x 3).

### Real-time PCR

FACS (Fluorescence activated cell sorting)-sorted prostate epithelial cells were lysed and total RNA was collected using RNeasy Plus kit (Qiagen). Synthesis of cDNA was performed using the iScript kit (BioRad) and real-time PCR carried out using SYBR green (Roche). Primer sequences were designed using Primer3 software and include the following: *Ar* (GGACCATGTTTTACCCATCG and TCGTTTCTGCTGGCACATAG), *Nkx3*.*1* (GACTGTGAACATAATCCAGGGG and CTCAGGGGCAGACAGGTACTT), *Tmprss2-ERG* (ATGGCATTGAACTCAGGGTCAC and GGCGTGGGGTGGCCGTGAC), and *Hprt* (TGCTCGAGATGTCATGAAGG and TATGTCCCCCGTTGACTGAT). Fold change in mRNA expression calculated using ΔΔCT method of values normalized to *Hprt*.

### FACS analysis/sorting and MACS

FACS analyses and sorting were performed as previously described [9]. Briefly, dissociated prostate epithelial cells were stained with specified fluorochrome-labeled antibodies (eBioscience) for 15 minutes on ice, washed, and analyzed/sorted using BD FACS Aria II flow cytometer. FACS analysis was performed using FlowJo CE software. Sorting based on Lineage (CD31, CD45, Ter119), Sca1, and CD49f was used to separate viable prostate epithelial cells from stroma [9].

### Data analysis

Statistical significance was calculated using the student t-test (real-time PCR & FACS data) and Chi-square test (pathology summaries) in GraphPad Prism. Analysis of human data was performed using cbioportal (www.cbioportal.org).

## Results and Discussion

### Genetic interaction between *Tmprss2-ERG* knockin and *Nkx3*.*1*-loss *in vivo* increases ectopic *ERG* expression in murine prostates

**Fig 1:**
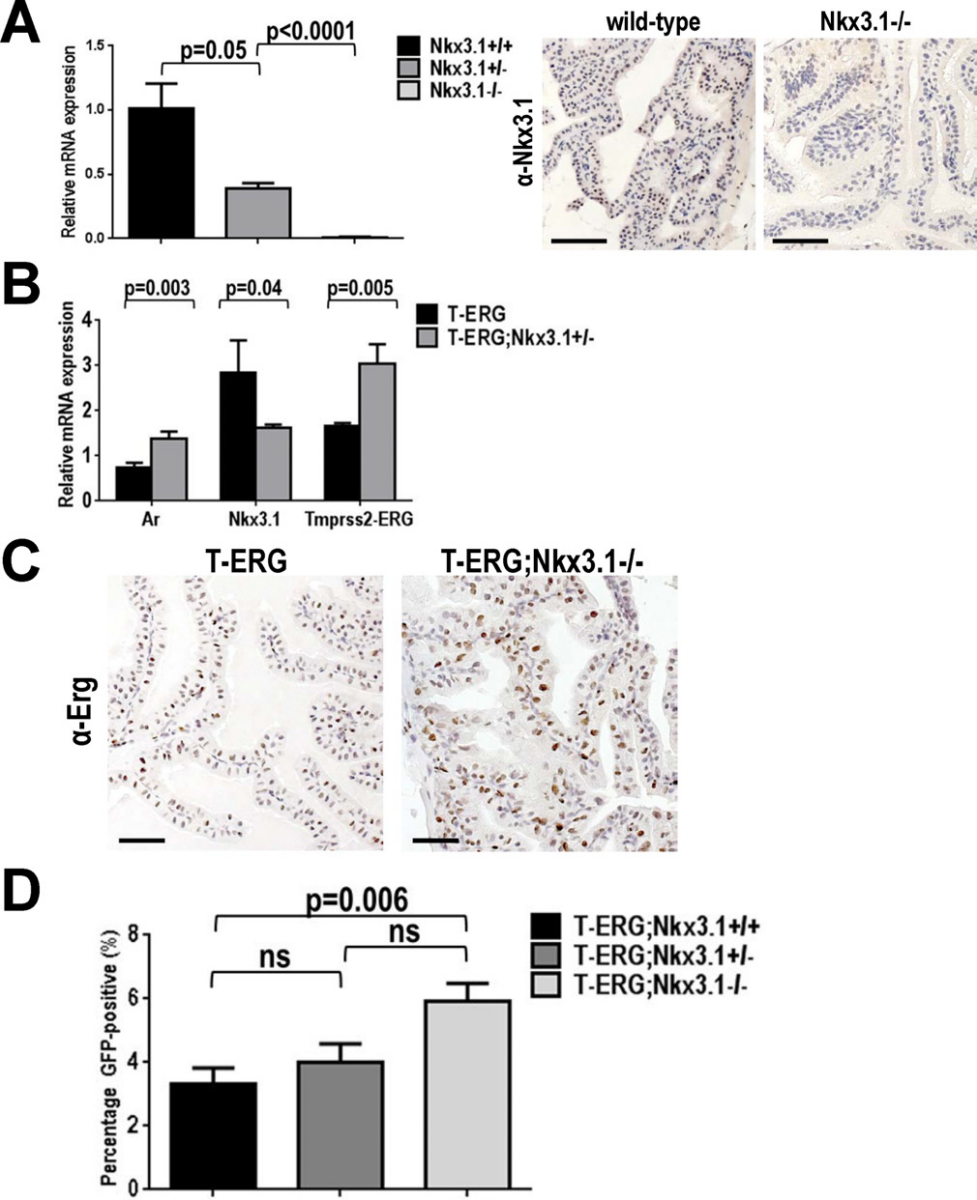
*Nkx3*.*1*-loss modestly increases the *Tmprss2* promoter activity *in vivo*. **A.** Progressive *Nkx3*.*1* transcript loss was confirmed in wild type (black) and heterozygous (dark gray) and homozygous (light gray) *Nkx3*.*1* knockout mice by real-time RT-PCR (left). Immunohistochemical (IHC) staining of anterior prostates (APs) using a mouse-specific Nkx3.1 antibody also validated Nkx3.1 protein loss. **B.** Real-time RT-PCR showing slight but statistically significant increase in the *Tmprss2-ERG* expression in *T-ERG;Nkx3*.*1* ^*+/-*^ double heterozygous males. **C.** IHC staining of APs showing increase in ectopic ERG expression at the protein level from the *T-ERG* knockin allele under the *Nkx3*.*1*-null background (*T-ERG;Nkx3*.*1* ^*-/-*^). H-scores were calculated as 81 and 127 for *T-ERG* and *T-ERG;Nkx3*.*1* ^*-/-*^ sections, respectively. **D.** FACS analysis showing progressive increase in the percentage of GFP^+^ cells in the prostates of *T-ERG;Nkx3*.*1* ^*+/-*^ and *T-ERG;Nkx3*.*1* ^*-/-*^ males, compared to those of males with *T-ERG* alone. Statistics: *p* values from Student t-test are indicated; ns = not significant. Scale bars represent 50 μm.

NKX3.1 is a critical regulator of prostate development and function and commonly exhibits loss of heterozygosity during human prostate cancer progression [13,14]. Mouse models of *Nkx3*.*1*-loss, however, do not develop overt prostate cancer and may only display evidence of epithelial hyperplasia or rare low-grade PIN (LG-PIN) lesions [17,20–22]. We first validated loss in both *Nkx3*.*1* transcript and protein expression in the prostates of mice carrying the *Nkx3*.*1* knockout allele (Fig. 1A). We then crossed *Nkx3*.*1* ^*+/-*^ mice to our *T-ERG* knockin mice [9] to generate *T-ERG;Nkx3*.*1* ^*+/-*^ and *T-ERG;Nkx3*.*1* ^*-/-*^ male mice.

In our *Tmprss2-ETS* knockin mouse models (i.e., *T-ERG*, *T-ETV1*), our strategy was to place the coding cDNAs of ETS transcription factors directly under the control of the endogenous murine *Tmprss2* promoter, thus accounting for androgen (and estrogen) regulation of this promoter [2,24], a critical feature of the *TMPRSS2-ETS* gene fusions that previous mouse models (mainly based on the *PB* promoter) have largely ignored [3–8]. This is especially relevant to *ETS* fusion biology given the role of AR signaling during prostate cancer progression and the fact that ERG can antagonize AR signaling [9,25]. Furthermore, a recent report demonstrated that NKX3.1 could negatively regulate the *TMPRSS2* locus through an evolutionary conserved NKX3.1 binding site within the *TMPRSS2* gene promoter upstream sequences, suggesting loss of *NKX3*.*1* could promote the *TMPRSS2-ERG* fusion expression in prostate cancer cells [19]. Since this NKX3.1 binding site is conserved between human and mouse [19], our *T-ERG* knockin model, which utilizes the endogenous *Tmprss2* control region to drive aberrant *ERG* expression, might be able to recapitulate it if this negative regulation indeed works *in vivo* under the physiological setting.

In *T-ERG;Nkx3*.*1* ^*+/-*^ double heterozygous males, in addition to the expected downregulation of the *Nkx3*.*1* transcript, we also observed a subtle but statistically significant increase in the *T-ERG* fusion transcript, which correlated with a concomitant increase in *Ar* expression levels (Fig. 1B). To further determine whether loss of *Nkx3*.*1* led to an increase in *T-ERG* expression at the protein level, we stained prostate sections from *T-ERG;Nkx3*.*1* ^*-/-*^ and control *T-ERG*-only males for ERG expression. By IHC staining and scoring, we indeed observed a notable increase in ERG protein level in the *T-ERG;Nkx3*.*1* ^*-/-*^ prostate (Fig. 1C). As such IHC scoring can be subjective, we utilized flow cytometry to more quantitatively measure GFP levels of prostate epithelial cells, as our *T-ERG* knockin allele carries an *ires-GFP* cassette introduced to the *Tmprss2* locus, which can be used as a surrogate for the transcription activity of both the endogenous *Tmprss2* and the *T-ERG* knockin fusion alleles [9]. We observed a slight but significant increase in GFP-positive (GFP^+^) prostate epithelial cells from *T-ERG;Nkx3*.*1* ^*-/-*^ prostates (compared to prostates from *T-ERG* only males), in line with our real-time PCR results (Fig. 1D). In addition, we also observed a slight increase in the mean fluorescent intensity (MFI) of GFP signals from *T-ERG;Nkx3*.*1* ^*-/-*^ prostates, though the increase did not reach statistical significance (S1 Fig.). Overall, these observations were consistent with the recent *in vitro* study demonstrating the negative regulation of the *TMPRSS2* locus by NKX3.1 [19]. Interestingly, ERG was found to repress *NKX3*.*1* expression [15], thus a feedback loop may exist between these commonly altered genes in prostate cancer. NKX3.1 was previously described to negatively regulate *AR* transcriptional activity as well as expression of *PSA*, another well established androgen-regulated gene [26,27]. Together these results confirm that the endogenous *Tmprss2* promoter activity is increased after *Nkx3*.*1*-loss, thereby resulting in a modest upregulation in *T-ERG* expression, and support that there is a genetic interaction between *TMPRSS2-ERG* gene fusion and *NKX3*.*1* (loss) in both murine models and human.

### *Tmprss2-ERG* knockin does not cooperate with *Nkx3*.*1*-loss *in vivo* to enhance prostate tumorigenesis

**Fig 2:**
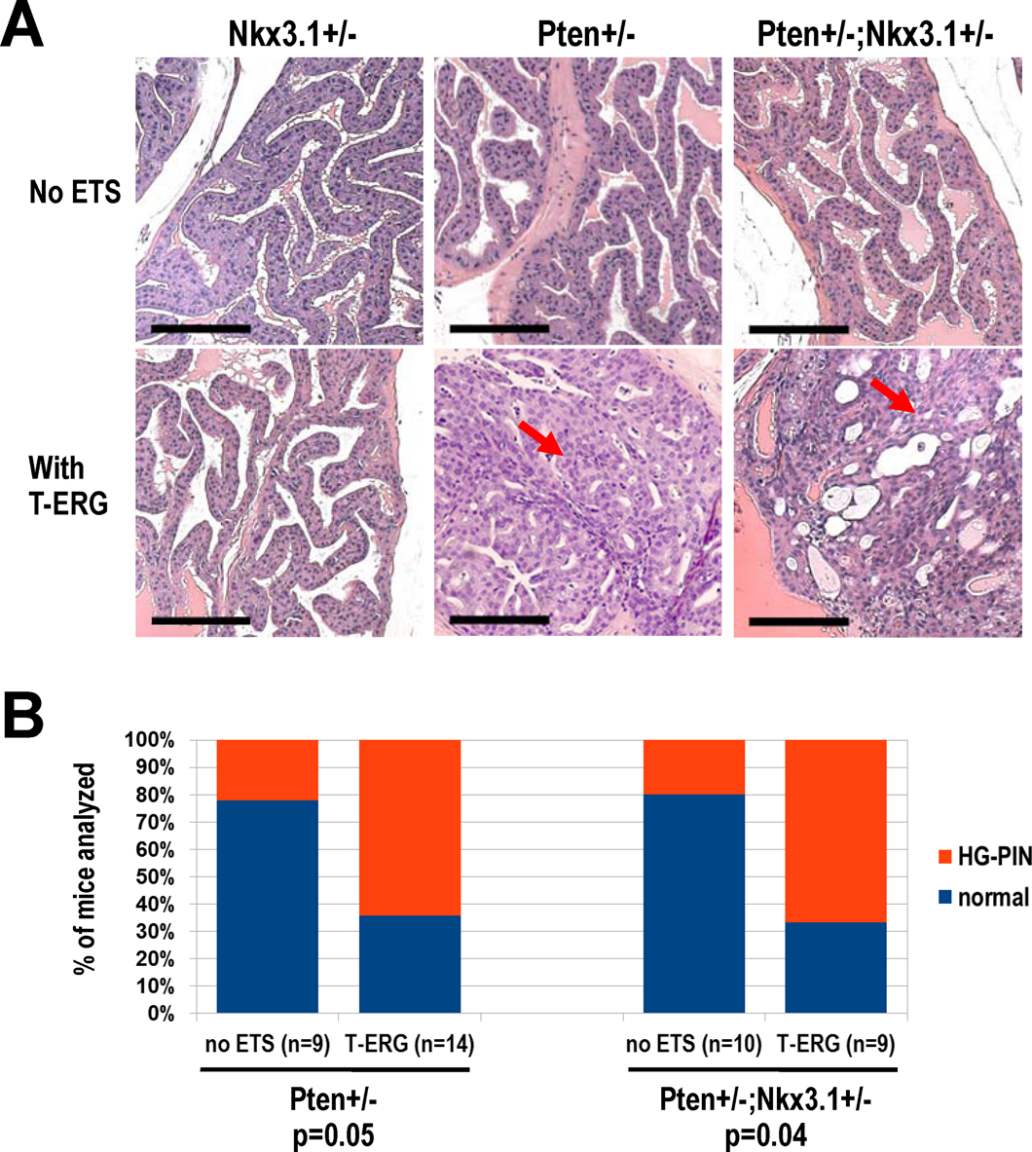
Heterozygous *Nkx3*.*1*-loss does not strongly cooperate with *Pten*-loss and *Tmprss2-ERG* expression. **A.** Representative anterior prostate (AP) histology of male mice with the indicated combinations of *Nkx3*.*1* ^*+/-*^, *Pten* ^*+/-*^, and *T-ERG* knockin. Note HG-PIN lesions developed in all prostate lobes of *T-ERG;Pten* ^*+/-*^ and *T-ERG;Pten* ^*+/-*^ *;Nkx3*.*1* ^*+/-*^ males due to cooperation between *Pten* ^*+/-*^ and *T-ERG*. Representative HG-PIN lesions developed in the APs of *T-ERG;Pten* ^*+/-*^ and *T-ERG;Pten* ^*+/-*^ *;Nkx3*.*1* ^*+/-*^ males are shown (red arrows). Scale bars represent 100 μm. **B.** Histology summary of aged *Pten* ^*+/-*^ (left) and *Pten* ^*+/-*^ *;Nkx3*.*1* ^*+/-*^ (right) male mice with or without the *T-ERG* knockin allele. Notable cooperation was detected with *T-ERG* (*p* = 0.05 under the *Pten* ^*+/-*^ background and *p* = 0.04 under the *Pten* ^*+/-*^ *;Nkx3*.*1* ^*+/-*^ background). HG-PIN in any prostate lobe was diagnosed by a trained rodent pathologist.

Despite the genetic interaction between *Tmprss2-ERG* gene fusion and *Nkx3*.*1*-loss even under the *Nkx3*.*1* ^*+/-*^ background (i.e., slight increase in *Tmprss2-ERG* expression, Fig. 1B, D), we did not observe any change in the prostate phenotype in *T-ERG;Nkx3*.*1* ^*+/-*^ double heterozygous males compared to *Nkx3*.*1* ^*+/-*^ single heterozygous males (Fig. 2A). In both *Nkx3*.*1* ^*+/-*^ and *T-ERG;Nkx3*.*1* ^*+/-*^ mice aged to at least 18 months of age, we found that their prostates were largely normal and rarely hyperplastic (Fig. 2A), with no signs of loss of heterozygosity. Interestingly, in our cohort, we also did not observe a significant cooperative effect between *Nkx3*.*1*-loss and *Pten*-loss in double heterozygote males (*Pten* ^*+/-*^ *;Nkx3*.*1* ^*+/-*^) compared to *Pten*-loss alone (*Pten* ^*+/-*^) control males, in terms of HG-PIN frequency, although we did observe the expected cooperativity between *T-ERG* knockin and *Pten*-loss (*Pten* ^*+/-*^) (which drives HG-PIN development, Fig. 2B). The mixed genetic background and dietary differences of our colony are likely contributors to the weaker phenotype compared to previously published reports (for *Pten* ^*+/-*^ *;Nkx3*.*1* ^*+/-*^) [18,28]. Not surprisingly, when under the *Pten* ^*+/-*^ *;Nkx3*.*1* ^*+/-*^ background, *T-ERG* mice exhibited a similar rate of cooperativity for driving development of HG-PIN lesions as that under the *Pten* ^*+/-*^ alone background (Fig. 2B), suggesting that loss of one copy of *Nkx3*.*1* does not further enhance the prostate cancer phenotype resulting from cooperation between *T-ERG* and *Pten* ^*+/-*^.

**Fig 3:**
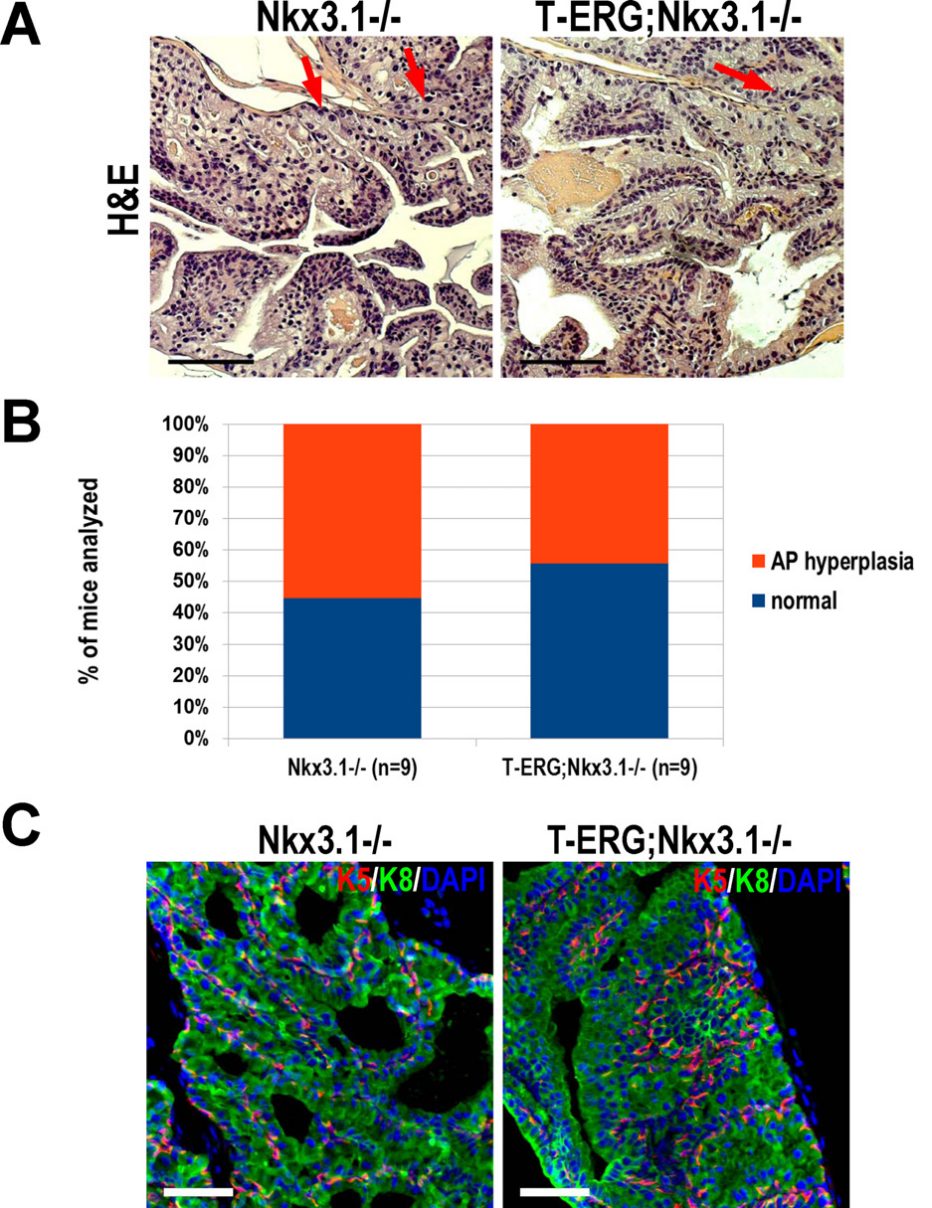
Total *Nkx3*.*1*-loss does not cooperate with *Tmprss2-ERG* gene fusion to promote prostate tumorigenesis. **A.** Representative anterior lobe (AP) histology of *Nkx3*.*1* ^*-/-*^ (left) and *T-ERG;Nkx3*.*1* ^*-/-*^ (right) mouse prostates stained with H&E. Scarce pleomorphic nuclei are evident (red arrows). Scale bars represent 100 μm. **B.** Graphical summary of histological findings of *Nkx3*.*1* ^*-/-*^ and *T-ERG;Nkx3*.*1* ^*-/-*^ male mice. There was no significant difference in AP hyperplasia frequency (*p* = 0.63). Histology was diagnosed by a trained rodent pathologist. **C.** IF staining for respective basal keratin 5 (K5, red) and luminal keratin 8 (K8, green) to visualize AP architecture in *Nkx3*.*1* ^*-/-*^ and *T-ERG;Nkx3*.*1* ^*-/-*^ mice. Nuclei counterstained with DAPI (blue). Scale bars represent 50 μm.

**Fig 4:**
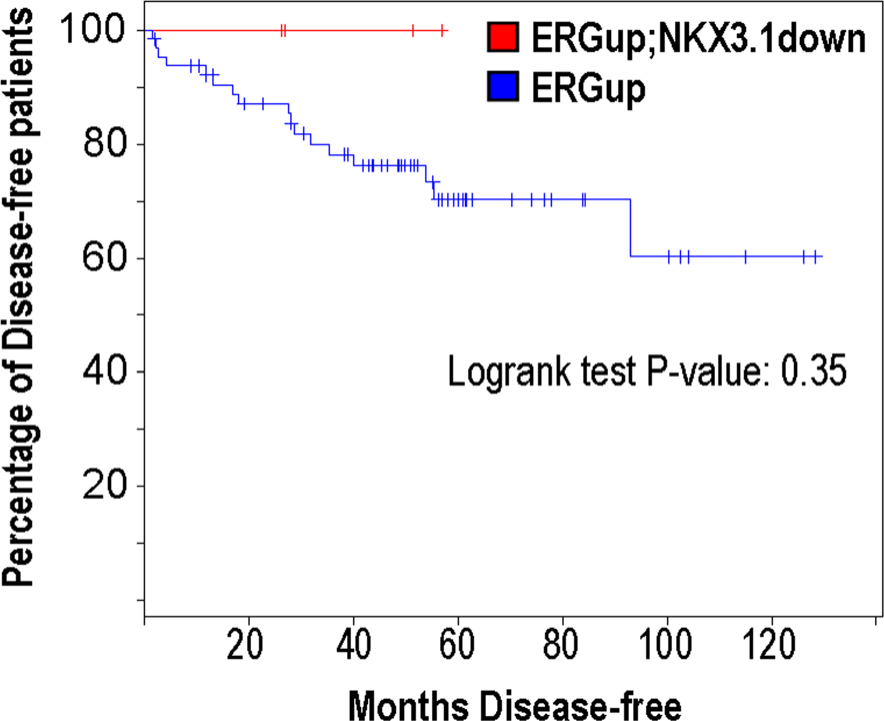
*NKX3*.*1*-loss in patients harboring *ERG* rearrangements is not predictive of biochemical relapse. Patient data from Taylor et al. [29] was used to compare via Kaplan-Meier analysis the disease-free survival of patients overexpressing *ERG*, which is highly predictive of harboring *TMPRSS2-ERG* fusion. Within this 'ERGup' cohort, patients who exhibited *NKX3*.*1* downregulation (red line, n = 4) compared to those who expressed normal levels of NKX3.1 (blue line, n = 65) were not more likely to display biochemical relapse. Logrank test *p* value was 0.35. Analysis performed using the cbioportal software [31].

As *Nkx3*.*1* ^*-/-*^ homozygous males exhibit a more severe phenotype than heterozygotes [16], we tested whether *Nkx3*.*1*-null would serve as a more sensitized background to test synergism with *ETS* fusions. Aged cohorts of *Nkx3*.*1*-null mice exhibited occasional diffuse pleomorphism and sparse patches of hyperplasia (Fig. 3A). Consistent with the previous reports, this phenotype was only notable in anterior prostate lobes (APs) with cribriform prostate proliferations histologically categorized between hyperplasia with atypia and LG-PIN (Fig. 3A) [16–18,20–22]. These prostates were often atrophic as well, indicating a perturbation in prostate development that is consistent with the known physiological role of Nkx3.1. Mice under the *Nkx3*.*1*-null background were phenotypically identical whether or not they harbored the *T-ERG* fusion (i.e., *Nkx3*.*1* ^*-/-*^ versus *T-ERG;Nkx3*.*1* ^*-/-*^) (Fig. 3A). Thus, despite the genetic interaction between these two events modestly increasing *T-ERG* expression, they do not appear to cooperate synergistically to a level that is sufficient to enhance prostate tumorigenesis. Although hyperplasia was common among these mice, a significant fraction of mice appeared histologically normal with only mild signs of atrophy in APs (Fig. 3A-B). No other lobes appeared affected by Nkx3.1 loss (data not shown). Furthermore, no disruption of basal or luminal epithelial layers was observed when analyzing keratins 5 and 8 (K5 and K8), respectively (Fig. 3C). We also analyzed an aged cohort of *Nkx3*.*1*-null mice which possessed the *T-ETV1* fusion (*T-ETV1;Nkx3*.*1* ^*-/-*^) and also did not observe evidence of cooperation (S2 Fig.). Lastly, an analysis of human prostate cancer data from Taylor et al. [29] revealed that within *ERG*-overexpressing prostate cancer patients, *NKX3*.*1* loss or deletion did not predict biochemical relapse after radical prostatectomy (Fig. 4). Notably the above-analyzed subpopulation from this cohort was small, thus further validation from a larger sample size is warranted. Overall, these results suggest that *Nkx3*.*1*-loss does not enhance the oncogenic effect of *ETS* fusions *in vivo*. These findings are in stark contrast to that of *Pten*-loss, in which mice with a single copy loss of *Pten* exhibit a dramatic increase in HG-PIN frequency and biallelic inactivation of *Pten* further accelerates invasive prostate cancer development [9]. Our negative results are in line with another published report utilizing a unique BAC construct to drive ERG expression from the endogenous human *TMPRSS2* control region, in the context of *Nkx3*.*1*-loss [30]. Thus, collectively, these studies suggest that *TMPRSS2-ETS* gene fusions display selective cooperation with other oncogenic perturbations (i.e., with *Pten*-loss, but not with *Nkx3*.*1*-loss).

Loss of *NKX3*.*1* and acquisition of *TMPRSS2-ETS* fusions are both frequent genetic alterations in human prostate cancer, and both events have been implicated in early prostate carcinogenesis [1,14]. In experimental models, neither of these alterations alone is strongly oncogenic, yet both readily cooperate with *Pten*-loss [8–10,18], suggesting that they serve to sensitize prostate cancer initiation rather than exert robust selective pressure during advanced disease progression. Our mouse modeling study further suggests that genetic interaction between these two common early events is also insufficient to drive prostate cancer progression. This observation may be explained by a possibility in which both events lead to a redundant molecular change in prostate cells (e.g., both *TMPRSS2-ERG* fusion and loss of *NKX3*.*1* may lead to a less differentiated state of prostate luminal cells [25,32,33]). Our data also suggests that *ETS* fusions like *TMPRSS2-ERG* are selective for which perturbations they cooperate with. This phenomenon was also observed in prostate regeneration assays where *ERG* overexpression cooperated with alterations in AR and PI3K signaling but not with *Trp53*-loss [11]. The precise mechanisms or pathways that *TMPRSS2-ERG* prefers exploit to promote prostate tumorigenesis remain largely elusive. As *ERG* overexpression itself does not appear to be prognostic for human prostate cancer progression (although some conflicting evidence in the literature exists [34]), further studies with larger cohorts and model systems may stratify clinical endpoints in patients harboring *ETS* gene fusions based on their cooperating oncogenic events.

## Supporting Information

S1 Fig *Nkx3*.*1*-loss modestly increases GFP expression from the *T-ERG* knockin allele harboring an *ires-GFP* reporter. **A.** Measurement of mean fluorescent intensity (MFI) of GFP signal from FACS showing a slight increase in the MFI of GFP from the *T-ERG* knockin allele when under the *Nkx3*.*1*-null background (when compared to that under the *Nkx3*.*1* wild type background), although the increase did not reach statistical significance (*p* = 0.08, ns = not significant). **B.** Representative FACS plots showing increase in GFP^+^ cells in the prostates of *T-ERG;Nkx3*.*1* ^*+/-*^ and *T-ERG;Nkx3*.*1* ^*-/-*^ males, compared to those of males with *T-ERG* alone.
(TIF)

S2 Fig *Nkx3*.*1*-loss does not cooperate with *Tmprss2-ETV1* expression. **A.** Representative histology of *T-ETV1;Nkx3*.*1* ^*-/-*^ and *Nkx3*.*1* ^*-/-*^ prostates in aged mice. H&E stained anterior prostate lobes are shown. Scale bar represents 100 μm. **B.** Graphical summary of histology results from all animals analyzed as shown in A. No significant cooperation with *T-ETV1* was detected (*p* = 0.34).
(TIF)
