# Supplementary figures and images for: Genetic Interaction between Tmprss2-ERG Gene Fusion and Nkx3.1-Loss Does Not Enhance Prostate Tumorigenesis in Mouse Models
Source: PLoS One. 2015 Mar 17;10(3):e0120628. doi: 10.1371/journal.pone.0120628 (PMC4364018; doi:10.1371/journal.pone.0120628)

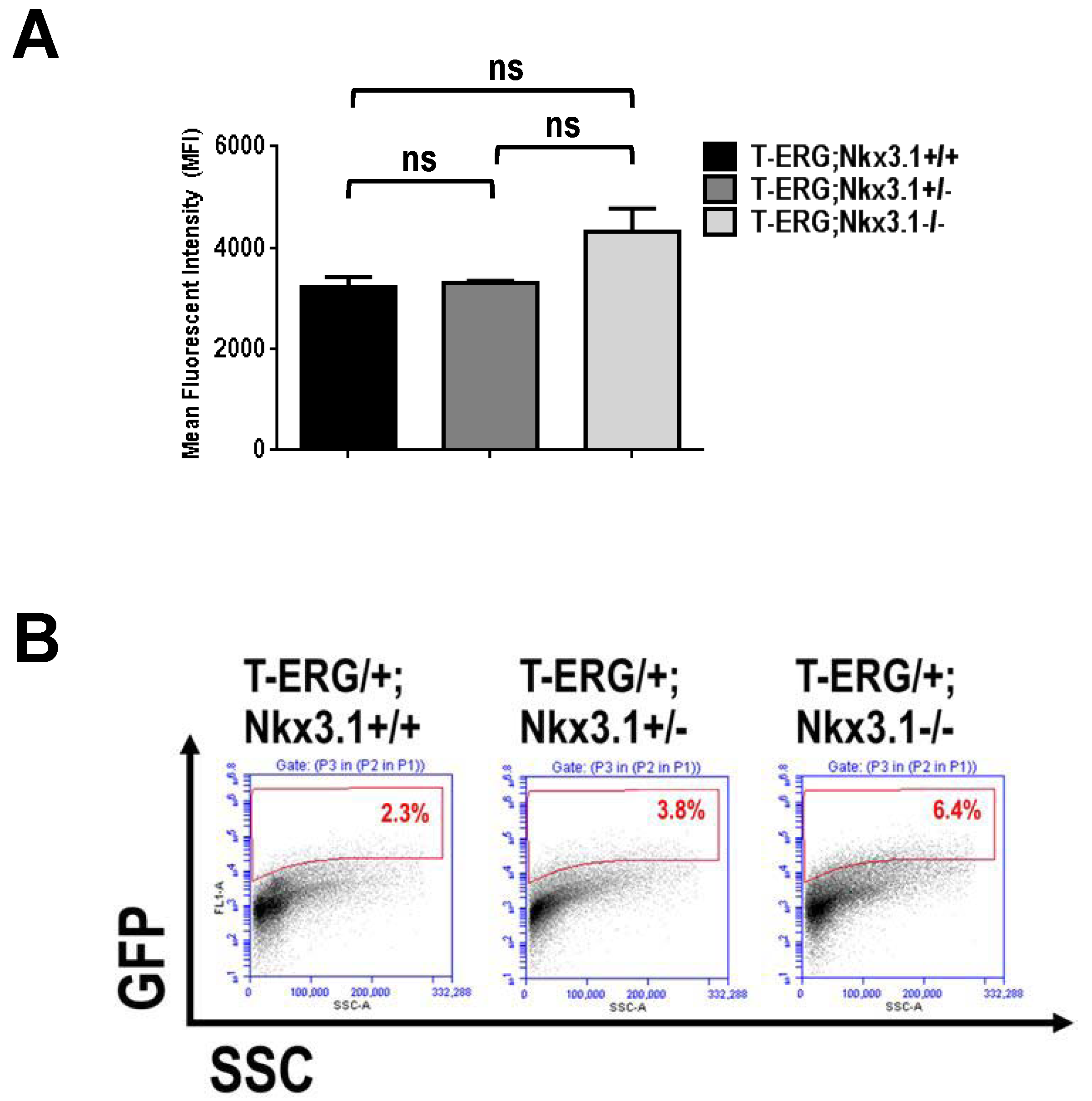

Supplement: S1 Fig — A. Measurement of mean fluorescent intensity (MFI) of GFP signal from FACS showing a slight increase in the MFI of GFP from the T-ERG knockin allele when under the Nkx3.1-null background (when compared to that under the Nkx3.1 wild type background), although the increase did not reach statistical significance (p = 0.08, ns = not significant). B. Representative FACS plots showing increase in GFP+ cells in the prostates of T-ERG;Nkx3.1 +/- and T-ERG;Nkx3.1 -/- males, compared to those of males with T-ERG alone. (TIF) [file pone.0120628.s001.tif]

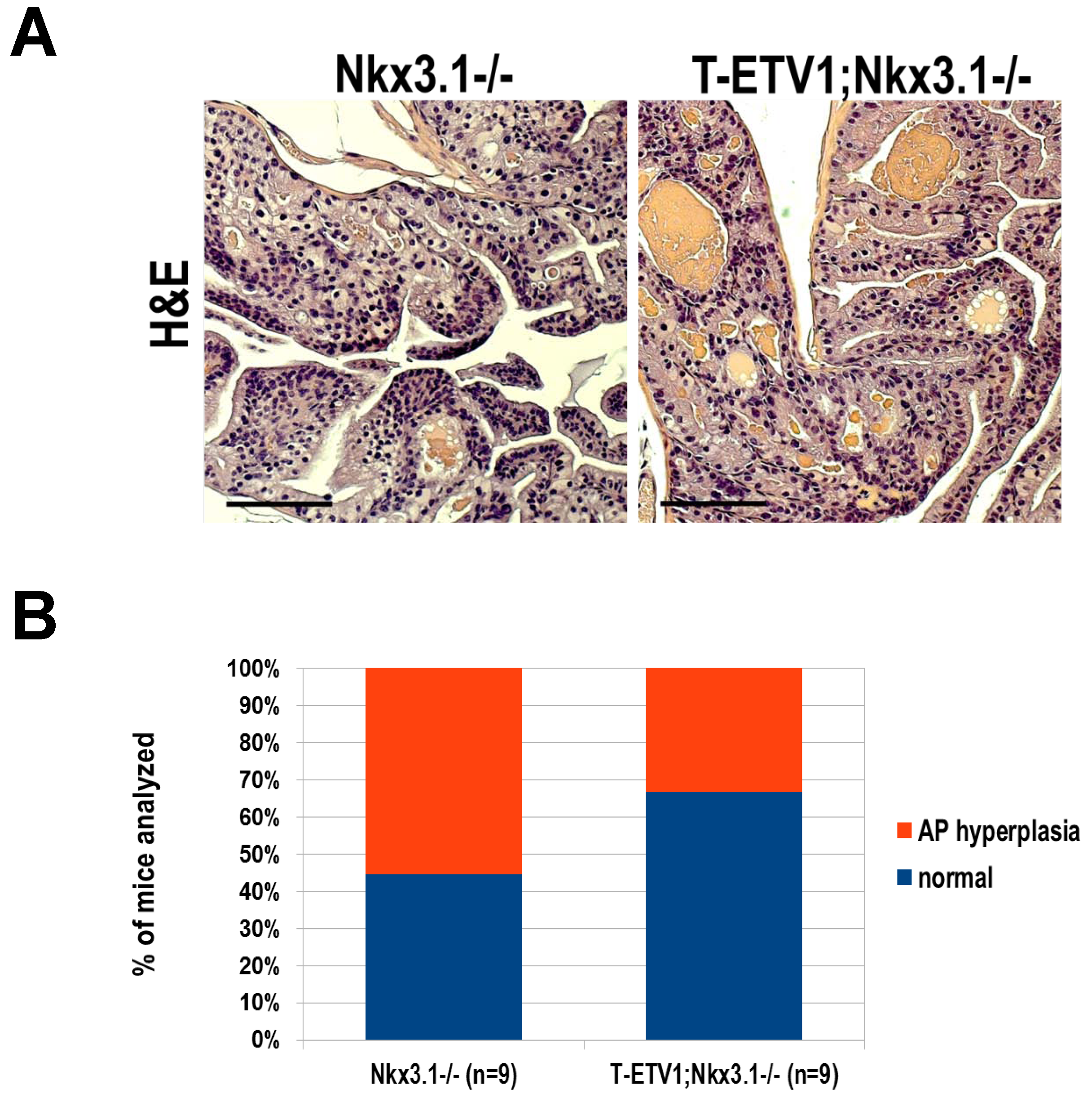

Supplement: S2 Fig — A. Representative histology of T-ETV1;Nkx3.1 -/- and Nkx3.1 -/- prostates in aged mice. H&E stained anterior prostate lobes are shown. Scale bar represents 100 μm. B. Graphical summary of histology results from all animals analyzed as shown in A. No significant cooperation with T-ETV1 was detected (p = 0.34). (TIF) [file pone.0120628.s002.tif]
